# Ameliorative Effect of N-Acetylcysteine Against 5-Fluorouracil-Induced Cardiotoxicity via Targeting TLR4/NF-κB and Nrf2/HO-1 Pathways

**DOI:** 10.3390/medicina61020335

**Published:** 2025-02-14

**Authors:** Omer Abdelbagi, Medhat Taha, Abdullah G. Al-Kushi, Mohammad Ahmad Alobaidy, Tourki A. S. Baokbah, Hatem A. Sembawa, Zohor Asaad Azher, Rami Obaid, Omar Babateen, Bayan T. Bokhari, Naeem F. Qusty, Hesham A. Malak

**Affiliations:** 1 Department of Pathology, Qunfudah Faculty of Medicine, Umm Al-Qura University, Al-Qunfudhah 28814, Saudi Arabia; omftab@hotmail.com; 2 Department of Anatomy, Al-Qunfudah Medical College, Umm Al-Qura University, Al-Qunfudhah 28814, Saudi Arabia; 3 Department of Anatomy and Embryology, Faculty of Medicine, Mansoura University, Mansoura 35516, Egypt; 4 Department of Anatomy, Faculty of Medicine, Umm Al-Qura University, Makkah 24382, Saudi Arabia; agkushi@uqu.edu.sa (A.G.A.-K.); maobaidy@uqu.edu.sa (M.A.A.); 5 Department of Medical Emergency Services, College of Health Sciences-AlQunfudah, Umm Al-Qura University, Al-Qunfudhah 28814, Saudi Arabia; tabaokbah@uqu.edu.sa; 6 Department of Surgery, Faculty of Medicine, Umm Al-Qura University, Makkah 24382, Saudi Arabia; hasembawa@uqu.edu.sa; 7 Department of Medical Genetics, Faculty of Medicine, Umm Al-Qura University, Makkah 24382, Saudi Arabia; zaazher@uqu.edu.sa; 8 Department of Medical Genetics, Faculty of Medicine at Al-Qunfudah, Umm Al-Qura University, Al-Qunfudhah 28814, Saudi Arabia; raaobaid@uqu.edu.sa; 9 Department of Physiology, Faculty of Medicine, Umm Al-Qura University, Makkah 24382, Saudi Arabia; ombabateen@uqu.edu.sa; 10 Department of Clinical Laboratory Sciences, Faculty of Applied Medical Sciences, Umm Al-Qura University, Makkah 24382, Saudi Arabia; btbokhari@uqu.edu.sa (B.T.B.); nfqusty@uqu.edu.sa (N.F.Q.); 11 Department of Biology, Faculty of Applied Science, Umm Al-Qura University, Makkah 24382, Saudi Arabia; hamalak@uqu.edu.sa; 12 Research Laboratories Centre, Faculty of Applied Science, Umm Al-Qura University, Makkah 24382, Saudi Arabia

**Keywords:** apoptosis, cardiotoxicity, inflammation, N-acetylcysteine, oxidative stress, 5-fluorouracil

## Abstract

*Background and Objectives*: 5-Fluorouracil (5-FU) is a widely prescribed and effective chemotherapeutic drug, but its cardiotoxic side effects pose a significant challenge to its use. Identifying a protective agent that does not affect its anticancer efficacy is essential. Our study aimed to investigate the cardioprotective effect of N-acetyl cysteine (NAC) against 5-FU-induced cardiac injury and to elucidate the underlying mechanisms. *Materials and Methods*: This study included four experimental groups, each with eight rats (*n* = 8): Group I (control group), Group II (NAC group), Group III (5-FU group), and Group IV (combined group 5-FU+NAC). Cardiac enzymes, oxidative stress, inflammatory, and apoptotic markers were investigated, and cardiac sections from the different groups were histologically examined. *Results*: Co-treatment of 5-FU with NAC resulted in significantly lower levels of cardiac enzymes (alanine transaminase (ALT) by 62.1%, aspartate transaminase (AST) by 73.6%, lactate dehydrogenase (LDH) by 55.8%, and creatine kinase (CK) by 57.3%) compared to the 5-FU group, along with marked improvements in heart tissue histology. Additionally, NAC enhanced the activity of cardiac antioxidant enzymes (superoxide dismutase (SOD) by 295.6%, catalase (CAT) by 181%, and glutathione peroxidase (GPx) by 320.9%) while decreasing malondialdehyde (MDA) by 51.1%, a marker of membranous lipid peroxidation. This might be due to significant upregulation of the nuclear factor erythroid-2-related factor 2 (Nrf2)/heme oxygenase-1 (HO-1) pathway at the gene and protein levels. The combined treatment significantly decreased the gene expression of the toll-like receptor 4 (TLR4)/nuclear factor kappa-light-chain-enhancer of activated B-cell (NF-κB) pathway. Furthermore, it downregulated the protein levels of inflammatory markers, including tumor necrosis factor-alpha (TNF-α) by 29.9%, interleukin-1 beta (IL-1β) by 21.9%, and interleukin-6 (IL-6) by 49.3%. Moreover, it upregulated the antiapoptotic marker B-cell lymphoma 2 (Bcl-2) protein levels by 269% and decreased apoptotic indicators Bcl-2-associated protein x (Bax) by 57.9% and caspase-3 by 30.6% compared to the 5-FU group. *Conclusions*: This study confirmed that NAC prevented the cardiotoxic effect of 5-FU through its antioxidant, anti-inflammatory, and antiapoptotic properties, suggesting its potential application as an adjuvant therapy in chemotherapy to alleviate 5-FU-induced cardiotoxicity.

## 1. Introduction

5-Fluorouracil (5-FU) is a potent chemotherapeutic drug commonly prescribed for various cancers, including breast, skin, and gastrointestinal tract cancers [1,2]. The systemic antitumor efficacy of 5-FU is attributed to its bioactive metabolites, such as fluorouridine triphosphate, fluorodeoxyuridine monophosphate, and fluorodeoxyuridine triphosphate. Fluorodeoxyuridine monophosphate inhibits DNA synthesis by binding to thymidine synthase, thereby blocking its function [3]. Administration of 5-FU results in the overproduction of reactive oxygen species (ROS), which promotes oxidation [4]. Although 5-FU has therapeutic benefits, it has several adverse effects, including emesis, nausea, mucositis, myelosuppression, and multiorgan toxicity, particularly affecting the heart [5]. 5-FU-induced cardiotoxicity is characterized by clinical manifestations, such as heart failure, arrhythmia, and myocardial ischemia, which present with symptoms including dyspnea, hypotension, and chest pain [6,7].

Toll-like receptor (TLR) activation mediates several pathways in innate immunity through inflammatory mediators such as myeloid differentiation factor 88 (MYD88) [8,9], which subsequently activates NF-κB, stimulating both M1 and M2 macrophages. M1 macrophages promote the secretion of inflammatory cytokines, while M2 macrophages produce IL-10 and IL-13, which are anti-inflammatory cytokines [10]. Previous research suggests that the TLR/MyD88 pathway plays a critical role in M1 polarization and the production of proinflammatory cytokines [10,11]. The release of interleukins, including IL-1β and IL-6, is believed to contribute significantly to cardiac injury induced by anticancer drugs. Therefore, controlling interleukin levels is essential for understanding 5-FU-induced cardiotoxicity [12]. The transcription factor Nrf2 is crucial in the antioxidant process by transcribing antioxidant enzymes through antioxidant response elements (AREs) [13,14]. Consequently, Nrf2 activation targets cytoprotective and antioxidant therapies [15,16,17].

N-acetylcysteine (NAC) is a mucolytic agent used to treat paracetamol toxicity [18]. L-cysteine, an NAC prodrug, is a precursor to glutathione (GSH). Regulation of GSH by NAC aids in maintaining the oxidation–antioxidation balance within cells, scavenging ROS, and downregulating lipid peroxidation [19,20]. NAC is considered a potent antioxidant against oxidative damage, both in vitro and in vivo, due to its role in regulating the oxidation–antioxidation balance [21,22]. Although several studies have discussed the beneficial effects of NAC in preventing chemotherapeutic-induced damage to renal, heart, and liver tissues [23,24,25], no research has investigated its protective effect against 5-FU-induced cardiac impairment and the associated molecular mechanisms. Therefore, this study was designed to examine the protective effect of NAC on 5-FU-induced cardiotoxicity in an experimental model, focusing on the associated molecular mechanisms, specifically the TLR4/NF-κB and Nrf2/HO-1 pathways.

## 2. Materials and Methods

### 2.1. Experimental Animals

Thirty-two male Wistar rats weighing 200–250 g were housed under suitable experimental conditions at 22 to 25 degrees Celsius, with a 12 h dark and 12 h light diurnal cycle. All animals had free access to food and water. All experimental measures were conducted following the protocols approved by the ethical committee of Umm Al-Qura University (Code number: HAPO-02-K-012-2024-06-2169).

### 2.2. Study Design

The rats in our study were divided into four equal experimental groups, with eight rats in each. Group I (control group, *n* = 8): rats in this group received normal saline for 14 days. Group II (NAC group, *n* = 8): rats in this group received N-acetylcysteine (purchased from the South Egypt Drug Industries Company (SEDICO), 6 October City, Egypt) at a dose of 200 mg/kg, injected intraperitoneally once daily for fourteen days [26,27]. Group III (5-FU group, *n* = 8): animals received 5-fluorouracil at a single dose of 100 mg/kg i.p. (from Sigma-Aldrich, St. Louis, MO, USA) on the first day of the experiment [28]. Group IV (5-FU+NAC, *n* = 8): rats in this group received both drugs at the same doses mentioned above and the same administration route.

### 2.3. Sample Collection

Twenty-four hours after the experiments, the rats from the different experimental groups were euthanized by cervical decapitation. Blood samples were collected for serum separation. Moreover, the hearts of the rats were removed and washed with ice-cold 50 mM Tris-HCl, pH = 7.4. The hearts were divided into three parts. The first part was homogenized in ice-cold 50 mM Tris-HCl, pH = 7.4, to produce a 10% homogenate (*w*/*v*), which was centrifuged for ten minutes at 3000× *g* at 4 degrees Celsius to obtain the supernatant for biochemical analysis. The second part, used for molecular analysis, was stored at −80 °C. Meanwhile, the third part was immersed in 10% buffered formalin for histological and immunohistochemical examination.

### 2.4. Evaluation of Cardiac Enzymes

The activities of CK, ALA, AST, and LDH were measured in the sera using commercial kits obtained from Spectrum Diagnostic (Cairo, Egypt), following the procedures established by Swanson and Wilkinson [29], Reitman and Frankel [30], and the International Federation of Clinical Chemistry and Laboratory Medicine (IFCC) [31], respectively.

### 2.5. Lipid Peroxidation and Antioxidant Enzymes Measurements

The cardiac supernatant level of the lipid peroxidation marker MDA was estimated following the method previously described by Buege and Aust [32]. The absorbance of MDA was read at 535 nm against a reagent blank, and the results were presented as nmol/g of cardiac tissue. GPx was measured using the method established by Beutler et al. [33], with results presented as nmol/g of tissue. CAT and SOD activities in the cardiac tissue supernatant were estimated following the procedures established by Aebi [34] and Minami and Yoshikawa [35], respectively. A four-score system was used to assess histopathologic findings, namely inflammation, hemorrhage, myocyte degeneration, and necrosis. The results were recorded as follows: 0 = 1–25%, 1 = 26–50%, 2 = 51–75%, and 3 = 76–100% [36].

### 2.6. Histopathological and Immunohistological Examination

For histopathological examination of cardiac tissues, the heart tissues were placed in 10% buffered formalin for 24 h. Following this, the cardiac tissues were washed with tap water and then immersed in a series of dilutions of ethyl alcohol. The cardiac specimens were embedded in paraffin blocks and sectioned into 5-micrometer-thick slices. These sections were stained with hematoxylin and eosin (H&E) and examined under light microscopy (Olympus CX31, Olympus, Tokyo, Japan) at 100× and 400× magnification [37].

The cardiac tissue sections were deparaffinized and dehydrated for immunostaining with graded ethyl alcohol. Afterward, the heart tissues were autoclaved for five minutes at 121 °C in distilled water for antigen retrieval. Next, the slides were immersed in 3% hydrogen peroxide to inactivate endogenous peroxidase. The slides were washed with PBS three times and then blocked with 5% bovine serum albumin for twenty minutes to reduce nonspecific reactions. The slides were incubated overnight at 4 °C with anti-caspase-3, anti-NF-κB, IL-1β, and Nrf2 antibodies (Cat# PA5-86276, PA5-27617, MBS2044253, and GTX103322). Subsequently, they were incubated at 37 °C with an avidin-biotin complex (ABC kit, Vector Laboratories Inc., Mowry Ave, CA, USA) for forty-five minutes. The immune reaction was visualized by combining with 3,3-diaminobenzidine tetrahydrochloride (DAB), and the cardiac slides were counterstained with Mayer’s hematoxylin.

The sections were labeled to avoid bias so that the observer could not identify them. Each captured image from each group was processed using the Fiji software 2.0.1. All images were converted to RGB for color adjustments. The percentage of positive area was analyzed using (http://fiji.sc) in eight sections from each group. A good contrast between DAB and hematoxylin in the field area was selected. Before capturing the image, color density and white balance were made constant. All captured images were saved in JPEG format. ImageJ software (Version 1.52f) was used to open the saved images using the bar. The color deconvolution function was applied, and the image was split into three colors. DAB staining, which was the second color, required adjustment of the image tool and the application of a threshold for IHC positive area selection. The area of positive staining was measured using the measurement tool. The average percentage from eight sections was calculated. All variables were expressed as mean ± standard deviation.

### 2.7. ELISA Examination of Proinflammatory and Apoptotic Markers

Inflammatory cytokines TNF-α, IL-6, Bax, and Bcl-2 in the cardiac supernatant were measured using ELISA kits (Cat# ab100785, ab234570, CSB-EL002573RA, and LS-F4135-1), following the manufacturer’s guidelines.

### 2.8. Quantitative Real-Time PCR (qRT-PCR)

Cardiac tissue RNA was extracted using the Qiagen RNeasy Plus Mini kit (Qiagen, Hilden, Germany). The purity and concentration of RNA samples were measured using a NanoDrop ND-100 spectrophotometer (NanoDrop Technologies, Wilmington, DE, USA), with absorption ratios at 260 nm and 260/280 nm. Two micrograms of RNA were reverse transcribed into cDNA using SuperScript III reverse transcriptase kits (Invitrogen, Carlsbad, CA, USA), following the manufacturer’s protocol and guidelines. Thermo Scientific (Waltham, MA, USA) Maxima™ SYBR Green/ROX qPCR Master Mix (2x) was used for amplification. Specific primers for the HO-1 gene were as follows: forward 5′-CATCCGTGCAGAGAATTCTG-3′ and reverse 5′-CTGGTATGGGCCCCACTGGC-3′; for the NFκB gene: forward 5′-GTCTCAAACCAAACAGCCTCAC-3′ and reverse 5′-CAGTGTCTTCCTCGACATGGAT-3′; for the Nrf2 gene: forward 5′-TCCCAAACAAGATGCCTTGT-3′ and reverse 5′-AGAGGCCACACTGACAGAGA-3′; for the MYD88 gene: forward 5′-TGGCGGAGGAGATGGGTTTCG-3′ and reverse 5′-AGCCTGCCGACCGACGAG-3′; for the TLR4 gene: forward 5′-CCGTCACCACATACTGCCTTTA-3′ and reverse 5′-GCAGTTTGGACTATTGAAATACGAAA-3′; and for the GAPDH housekeeping gene: forward 5′-CCTCGTCTCATAGACAAGATGGT-3′ and reverse 5′-GGGTAGAGTCATACTGGAACATG-3′.

Initial denaturation during real-time PCR was performed at 94 °C for one minute, followed by 40 amplification cycles (denaturation at 94 °C for ten seconds, annealing at 60 °C for thirty seconds, and extension at 72 °C for sixty seconds). The threshold cycle (Ct) for target genes was normalized to the mean critical threshold value of the GAPDH housekeeping gene. According to Livak and Schmittgen [38], gene expression quantification was determined using the Ct ^2−ΔΔ^ equation method.

### 2.9. Statistical Analysis

The results of our work were presented as mean ± SD. A one-way analysis of variance (ANOVA) followed by Tukey’s post hoc test was performed using GraphPad Prism (version 8.0; La Jolla, CA, USA), with *p* < 0.05 considered significant.

## 3. Results

### 3.1. Effect of NAC on Cardiac Enzyme Levels and Histopathological Alternation

**Figure 1:**
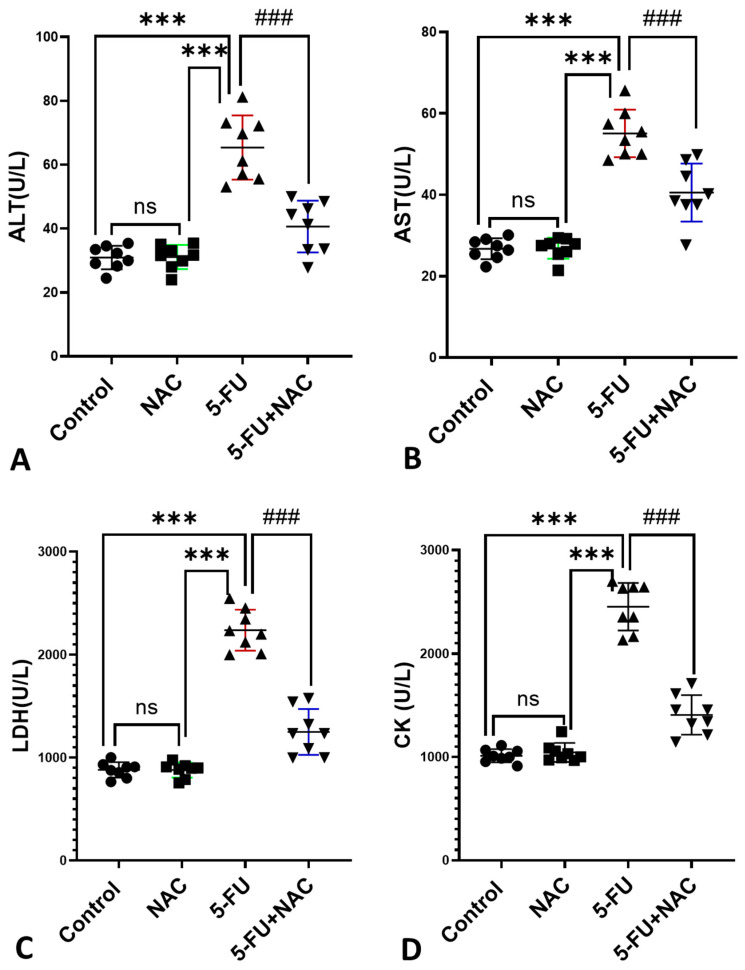
Effect of NAC on cardiac enzymes: (**A**) ALT, (**B**) AST, (**C**) LDH, and (**D**) CK. All data are presented as mean ± SD. *** *p* < 0.001 indicates significance compared to control and NAC rats, and ^###^ *p* < 0.001 indicates significance compared to the 5-FU rats’ group, ns, non-significant.

As shown in Figure 1, 5-FU intraperitoneal injection caused cardiac impairment, evidenced by a significant increase in the serum levels of ALT, AST, LDH, and CK by 211.2%, 205.9%, 254%, and 242.5%, respectively, compared to the control and NAC groups (Figure 1A–D, *p* < 0.001). However, coadministration of NAC with 5-FU resulted in a significant (*p* < 0.001) decrease in ALT, AST, LDH, and CK by 62.1%, 73.6%, 55.8%, and 57.3%, respectively, compared to the 5-FU group. Additionally, no significant difference was detected between control rats and those treated with NAC alone. Based on these results, NAC has a protective role against 5-FU-induced cardiac injury.

**Figure 2:**
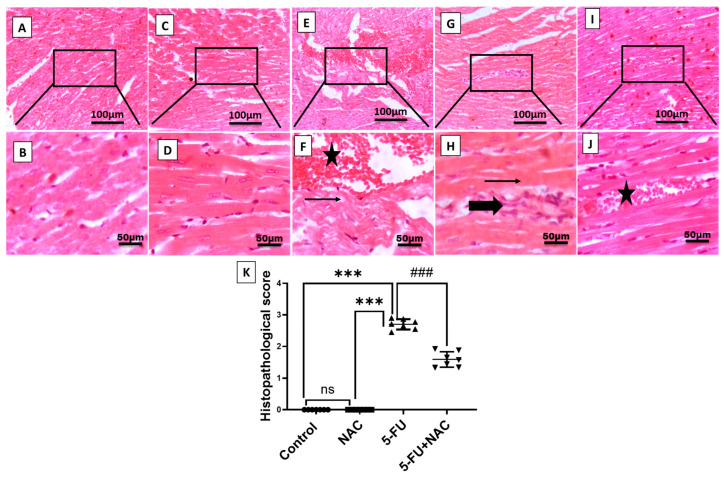
Representative photomicrographs of heart sections: (**A**–**D**) control and NAC groups show a normal histological appearance of cardiac myocytes; (**E**,**F**) 5-FU group displays severe hemorrhage, with widely separated myocytes replaced by myocardial degeneration and necrosis; (**G**,**H**) 5-FU group demonstrates focal inflammation surrounding myocardial necrosis with shrunken, hypereosinophilic sarcoplasm; (**I**,**J**) 5-FU+NAC group shows focal areas of hemorrhage. (**K**) histopathological scoring. Thin arrow = myocardial necrosis, thick arrow = inflammation, star = hemorrhage. Image magnification = 100× and 400×. All data are presented as mean ± SD. *** *p* < 0.001 indicates significance compared to control and NAC rats, and ^###^ *p* < 0.001 indicates significance compared to the 5-FU rats’ group.

Consistently, histopathological examination of heart tissues from different experimental groups revealed variations. The control and NAC groups exhibited a normal appearance of cardiac myocytes (Figure 2A–D). In contrast, the 5-FU group showed myocardial degeneration, characterized by necrotic myocytes with hypereosinophilic cytoplasm, severe hemorrhage, and focal inflammation (Figure 2E–H). Group IV, which received both NAC and 5-FU treatments, notably demonstrated marked improvement in cardiac tissue, with only focal hemorrhage observed (Figure 2I,J). These histological changes in the different groups were confirmed through the histological scoring (Figure 2K). The histological improvement in cardiac tissues due to NAC supports its cardioprotective effect on serum levels of cardiac enzymes.

### 3.2. Effect of NAC on 5-FU-Induced Oxidative Stress

**Figure 3:**
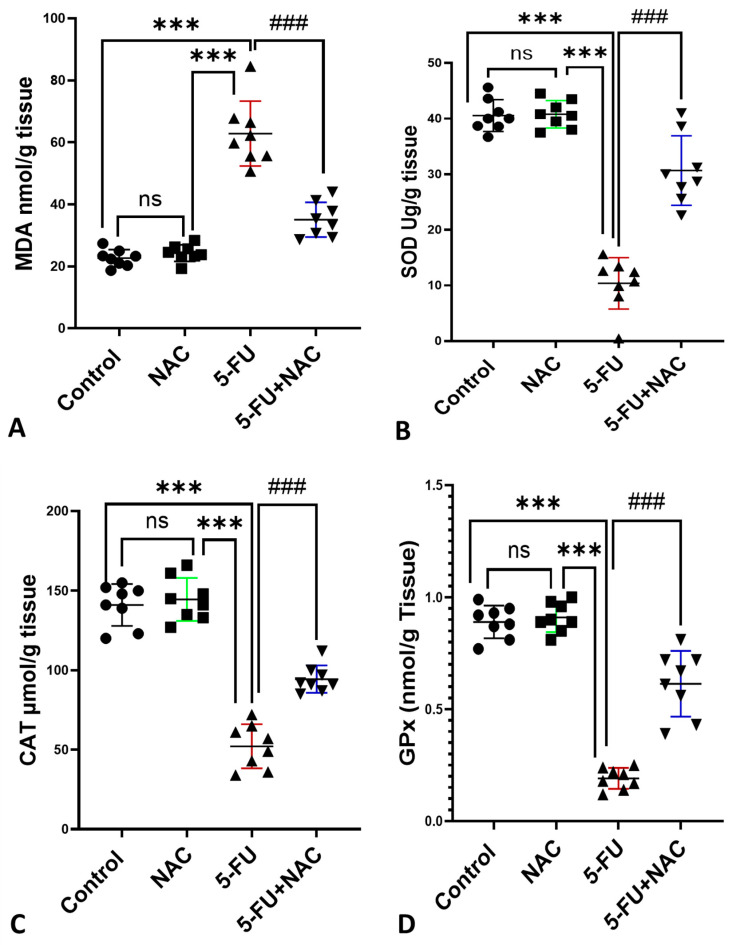
Effect of NAC on (**A**) lipid peroxidation indicator MDA and antioxidant enzymes (**B**) SOD, (**C**) CAT, and (**D**) GPx in cardiac supernatant. All values are expressed as mean ± SD. *** *p* < 0.001 indicates significance compared to control and NAC rats, and ^###^ *p* < 0.001 indicates significance compared to the 5-FU rats’ group. ns, non-significant.

As oxidative stress is involved in the pathology of 5-FU-induced cardiac damage, the results of oxidative stress markers MDA, SOD, CAT, and GPx are presented in Figure 3A–D. Group III (5-FU group) showed a significant increase in MDA by 380.3% compared to the control and NAC groups (*p* < 0.001). Meanwhile, coadministration of NAC with 5-FU decreased the cardiac homogenate MDA by 51.1% compared to the 5-FU group. Conversely, the levels of antioxidant enzymes SOD, CAT, and GPx significantly decreased by 25.6%, 36.9%, and 21.4%, respectively, in the 5-FU group compared to the control and NAC groups (*p* < 0.001). In contrast, NAC co-treatment with 5-FU resulted in significant increases in the levels of SOD, CAT, and GPx by 295.6%, 181%, and 320.9%, respectively, compared to the 5-FU group (*p* < 0.001).

**Figure 4:**
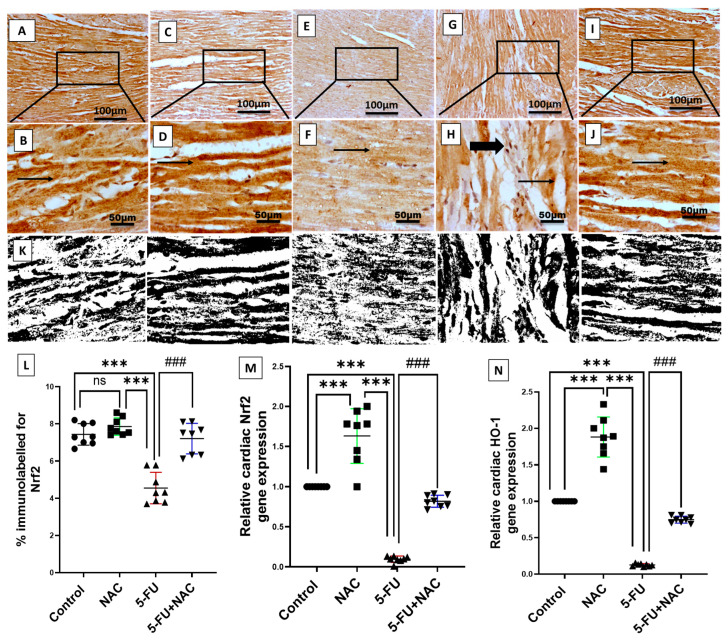
Photomicrographs of Nrf2 immunoexpression in cardiac sections of all groups: (**A**,**B**) Normal group shows highly cytoplasmic-stained myocytes; (**C**,**D**) NAC group showing highly immunostained myocytes; (**E**,**F**) 5-FU group displays mild to moderate positive cytoplasmic staining in myocytes; (**G**,**H**) 5-FU group shows moderate cytoplasmic immunopositive staining in myocytes with mild staining of inflammatory cells; (**I**,**J**) 5-FU+NAC group demonstrates highly immunostained myocytes. Image magnification = 100× and 400×. The thin arrow indicates positive myocytes, while the thick arrow indicates positive inflammatory cells. (**K**) Image analysis of Nrf2 expression in different treatment groups. (**L**) Quantification of Nrf2 percentage expression in cardiac tissue was performed using ImageJ software (Version 1.52f). (**M**,**N**) Gene expression of Nrf2 and HO-1. All results are expressed as mean ± SD. *** *p* < 0.001 vs. control and NAC rats, and ^###^ *p* < 0.001 vs. the 5-FU rats’ group. ns, non-significant.

Nrf2/HO-1 pathway activation plays a crucial role in the transcription of antioxidant enzymes. Therefore, we investigated the cardiac expression of this pathway. The 5-FU group exhibited a marked decrease in the immunoexpression and mRNA levels of Nrf2 and gene expression of HO-1 by 61.3%, 90%, and 120%, respectively, compared to the control and NAC groups (Figure 4E–H,L–N, *p* < 0.001). In contrast, NAC treatment with 5-FU in Group IV significantly improved the Nrf2/HO-1 pathway by 158.2%, 900%, and 616.6%, respectively, compared to the 5-FU group (Figure 4I,J,L–N, *p* < 0.001). There is no significant difference between the control and NAC groups in the above parameters, except for the mRNA levels of Nrf2 and HO-1, where the NAC-alone-treated animals showed significant differences compared to the control animals. From the above, NAC demonstrates a powerful antioxidant effect against 5-FU-induced cardiotoxicity.

### 3.3. NAC Decreased Inflammatory Markers Production in 5-FU-Induced Cardiotoxicity

**Figure 5:**
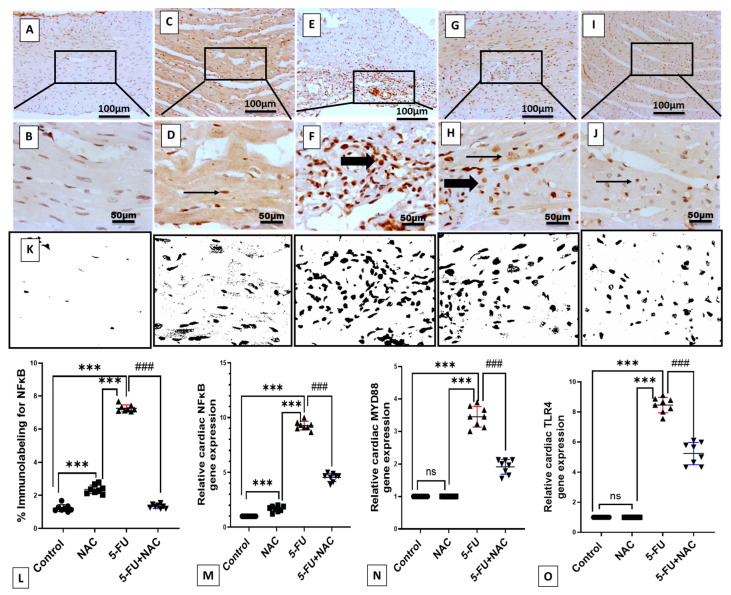
Representative immunohistochemistry (IHC) of NF-κB expression in different cardiac sections. (**A**,**B**) The normal group shows negative nuclear staining in myocytes. (**C**,**D**) The NAC group demonstrates minimal nuclear immunostaining in myocytes. (**E**,**F**) 5-FU group exhibits strong nuclear staining in invading inflammatory cells. (**G**,**H**) 5-FU group demonstrates high nuclear positivity in both myocytes and inflammatory cells. (**I**,**J**) The 5-FU+NAC group displays mild faint nuclear staining in myocytes. Image magnification = 100× and 400×. The thin arrow indicates positive myocytes, while the thick arrow indicates positive inflammatory cells. (**K**) Image analysis of NF-κB expression in heart sections. (**L**) Histogram of NF-κB percentage immunolabeling. (**M**–**O**) Gene expression of NF-κB, TLR4, and MYD88. All values are presented as mean ± SD, *** *p* < 0.001 versus the control and NAC groups, ^###^ *p* < 0.001 versus the 5-FU rats’ group. ns, non-significant.

**Figure 6:**
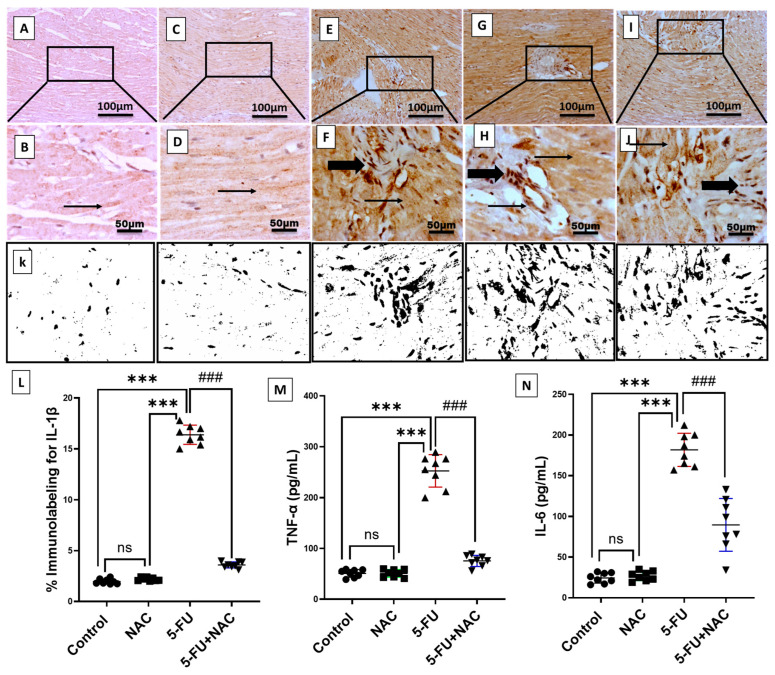
Immunohistochemical staining of IL-1β in cardiac sections of all groups. (**A**,**B**) The normal group shows faint immunopositive staining in myocytes. (**C**,**D**) The NAC group demonstrates faint immunostaining in myocytes. (**E**,**F**) 5-FU group shows strong sarcoplasmic positivity with interstitially positive stained inflammatory cells. (**G**,**H**) 5-FU group exhibits extensive sarcoplasmic staining along with cytoplasmic and nuclear immunopositivity in inflammatory cells. (**I**,**J**) 5-FU+NAC group shows moderate cytoplasmic expression in myocytes with few positively stained inflammatory cells. Image magnification = 100× and 400×. The thin arrow indicates positively stained myocytes, while the thick arrow indicates positively stained inflammatory cells. (**K**) Images of IL-1β expression analysis in heart sections. (**L**) Histogram of IL-1β immunolabeling percentage. (**M**,**N**) Protein levels of TNF-α and IL-6 in different groups. All results are presented as mean ± SD, *** *p* < 0.001 indicates significance compared to control and NAC animals, ^###^ *p* < 0.001 indicates significance compared to the 5-FU rats’ group. ns, non-significant.

The levels of inflammatory markers in cardiac tissues can be seen in Figure 5 and Figure 6. Following intraperitoneal injection of 5-FU, there was a significant increase in the immunoexpression and mRNA levels of NF-κB, as well as the gene expression of TLR4 and MYD88, by 590.2%, 926%, 845.6%, and 344%, respectively, compared to control and NAC rats (Figure 5E–H,M–O, *p* < 0.001). Furthermore, the immunoexpression of IL-1β and the protein levels of TNF-α and IL-6 increased by 831.4%, 494.9%, and 745.7%, respectively, compared to control and NAC animals (Figure 6E–H,M,N, *p* < 0.001). However, NAC co-treatment significantly downregulated the levels of inflammatory markers NF-κB, TLR4, MYD88, IL-1β, TNF-α, and IL-6 by 18.3%, 49.1%, 61.8%, 55.5%, 21.9%, 29.9%, and 49.3%, respectively, compared to the 5-FU rats (Figure 5I,J,M–O and Figure 6I,J,M,N, *p* < 0.001). In most of the previous parameters, no significant difference was observed between the control and NAC-alone groups, except for NF-κB immunoexpression and mRNA levels, where the NAC-alone-treated group showed a high significance (*p* < 0.001) compared to the control group. These findings suggest that NAC exhibits a strong anti-inflammatory effect against 5-FU-induced cardiac inflammation.

### 3.4. Ameliorative Effect of NAC Against 5-FU-Induced Cardiac Apoptosis

**Figure 7:**
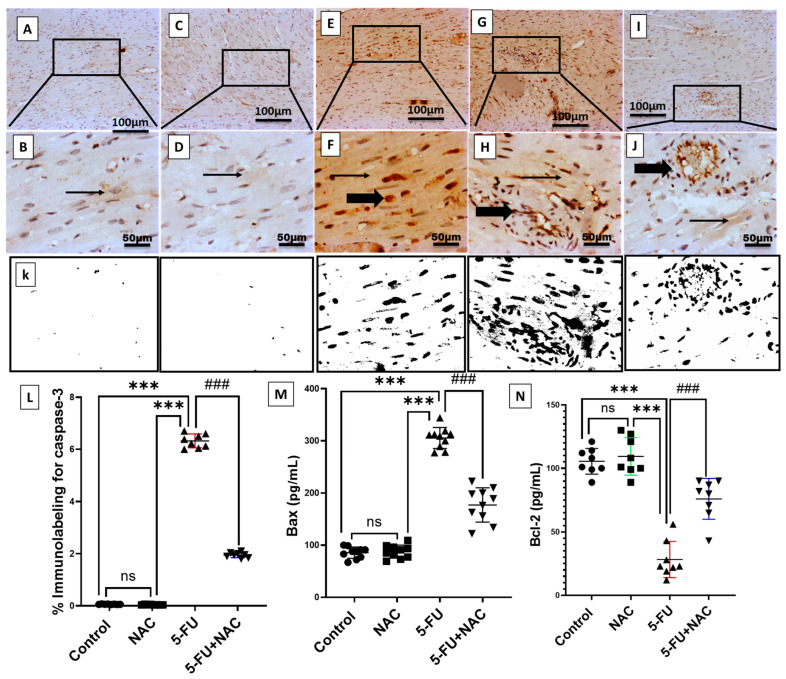
Caspase-3 immunostaining in cardiac sections from different experimental groups. (**A**,**B**) Normal rat sections show negative to faint immunopositive staining in myocytes. (**C**,**D**) NAC group exhibits negative to faint immunostaining in myocytes. (**E**,**F**) 5-FU group demonstrates high positive staining in the sarcoplasm along with positively stained inflammatory cells. (**G**,**H**) 5-FU group shows significant immunopositive staining in perivascular inflammatory cells with sarcoplasmic positivity. (**I**,**J**) The 5-FU+NAC group illustrates weak cytoplasmic expression in myocytes and perivascular inflammatory cells. Image magnification = 100× and 400×. The thin arrow indicates positively stained myocytes, while the thick arrow indicates positively immunostained inflammatory cells. (**K**) Image analysis of caspase-3 expression in cardiac sections from different treatment groups. (**L**) Quantification of caspase-3 percentage expression in cardiac tissue. (**M**,**N**) Protein levels of TNF-α and IL-6 in different groups. All data are presented as mean ± SD, with *** *p* < 0.001 indicating significance compared to control and NAC rats and ^###^ *p* < 0.001 indicating significance compared to the 5-FU rats’ group. ns, non-significant.

To explore the underlying mechanisms involved in the protection provided by NAC against 5-FU-induced cardiac injury, the levels of cardiac apoptotic markers, including caspase-3, Bax, and the antiapoptotic protein Bcl-2 were investigated through immunoexpression and protein assays. Figure 7 demonstrates a significant elevation in the immunoexpression of caspase-3 and the ELISA levels of Bax, which increased by 1053% and 356.5%, respectively, compared to the control and NAC group (Figure 7E–H,L,M, *p* < 0.001). Conversely, 5-FU administration decreased the antiapoptotic marker Bcl-2 by 26.7% compared to control animals (Figure 7I,J,N). In contrast, combined treatment with NAC and 5-FU effectively reduced cardiomyocyte apoptosis, restoring the levels of caspase-3, Bax, and Bcl-2 by 30.6%, 57.9%, and 269%, respectively, compared to the 5-FU group. Notably, no significant difference existed between the control and NAC alone-treated groups. These findings clarify the antiapoptotic effect of NAC against 5-FU-induced cardiomyocyte apoptosis.

## 4. Discussion

5-Fluorouracil (5-FU), a chemotherapeutic agent, induces toxicity in multiple organs [39]. Its cardiotoxic effects are well established, with several cases of myocardial infarction, coronary spasm, and arrhythmia reported [40]. NAC, a well-known mucolytic agent, possesses potent antioxidant properties through its induction of glutathione [41]. Numerous studies have highlighted its anti-inflammatory properties and antioxidant effects, leading to its examination in several inflammatory and toxic models [41,42]. This study aimed to examine the cardioprotective effect of NAC against 5-FU-induced cardiac injury and elucidate its underlying mechanisms.

Overall, the findings of the present study demonstrated that NAC reduces serum levels of ALT, AST, LDH, and CK, thereby minimizing cardiac injury. This is evidenced by improvements in the histological appearance of cardiac tissues. Additionally, NAC mitigates cardiac oxidative stress, inflammation, and apoptosis in 5-FU-induced cardiac impairment.

In this study, 5-FU induced significant cardiac injury, as evidenced by a marked elevation in ALT, AST, LDH, and CK serum levels. These enzymes are abundant in cardiac tissues and indicate cardiac degeneration. This finding aligns with a previous study by Numan et al. [43], which reported that 5-FU administration elevates ALT, AST, and CK levels in a rat model. The elevation of these cardiac enzymes indicates myocardial injury, characterized by significant leakage of these enzymes. Conversely, intraperitoneal injection of NAC significantly reduced serum levels of cardiac enzymes, consistent with previous studies [24,26]. The improvement in the histological appearance of cardiac tissues in Group IV can be attributed to NAC’s inhibitory effect on cardiac enzymes, serving as an indicator of reduced cardiac damage.

Several mechanisms are involved in the pathology of 5-FU-induced cardiac injury, including oxidative stress and the production of free radicals, which activate intrinsic and extrinsic apoptotic pathways linked to caspase family activation [44,45,46,47]. The disruption of oxidative balance leads to an imbalance between prooxidant and antioxidant production, resulting in ROS accumulation, which causes irreversible damage to macromolecules, nucleic acids, and DNA [48]. Our results showed that intraperitoneal injection of 5-FU increased MDA levels while decreasing SOD, CAT, and GPx levels, which are indicators of lipid peroxidation and the intrinsic antioxidant enzyme system. This finding is supported by the work of Karim et al. [49]. However, co-treatment with NAC significantly elevated antioxidant enzyme levels and decreased MDA levels, consistent with the findings of Demir et al. [50], who documented the antioxidant effect of NAC against 5-FU-induced ovarian damage.

Nrf2, a transcription factor, plays a regulatory role in cellular oxidative damage. During oxidative stress, Nrf2 translocates into the nucleus after dissociating from Keap1, activating the production of antioxidant enzymes and maintaining cellular redox homeostasis [51]. In the current study, data revealed that 5-FU markedly downregulates Nrf2/HO-1 pathway immunoexpression and gene expression, aligning with the results of Lokman et al. [52]. Meanwhile, the NAC co-treated group significantly upregulates the Nrf2/HO-1 pathway compared to the 5-FU group, supported by the findings of Khanna et al. [53], which reported the enhanced effect of NAC on the Nrf2 pathway in cigarette-induced myocardial infarction, reducing cardiac oxidative stress. The beneficial antioxidant effect of NAC in our study against 5-FU-induced cardiac impairment can be attributed to its enhancement of glutathione production, which in turn stabilizes Nrf2 and promotes its nuclear translocation. Furthermore, NAC may modulate other upstream regulators, such as p62/SQSTM1, which interacts with Keap1 and facilitates Nrf2 activation, enhancing the antioxidant response. Additionally, the involvement of alternative pathways, such as the PI3K/Akt signaling pathway, which has been shown to regulate Nrf2 activity positively, might contribute to NAC’s cardioprotective effects. Studies have demonstrated that NAC can activate the PI3K/Akt pathway, leading to enhanced Nrf2 activation and subsequent upregulation of antioxidant defenses [54,55]. Moreover, NAC exerts a direct effect on antioxidant enzyme production.

Furthermore, NAC’s antioxidant properties explain its cardioprotective effect by preventing oxidative-induced cardiomyocyte membrane damage and the leakage of cardiac enzymes into the serum [56,57,58].

It is well established that cardiac inflammation is involved in 5-FU-induced cardiotoxicity. TLRs are a subfamily of recognition receptors that mediate innate and adaptive immunity [59,60]. TLRs play a role in various inflammatory and apoptotic disorders by producing inflammatory cytokines. For example, TLR5 can recognize several pathogen-associated molecules, releasing profibrotic factors and inflammatory cytokines, possibly resulting in myocardial infarction. Studies have shown that a deficiency in TLR5 reduces oxidative stress and inflammation, thereby minimizing cardiac fibrosis and dysfunction [61]. 5-FU induces an inflammatory response characterized by producing proinflammatory mediators that activate TLRs, including TLR2 and TLR4. This activation triggers the downstream NF-κB pathway, producing TNF-α, IL-1β, and IL-6 inflammatory cytokines [62,63,64]. These cytokines promote programmed cell death by disrupting the balance between apoptotic and antiapoptotic factors, increasing the release of various caspases, including caspase-3, a well-known marker of cellular apoptosis [61,62].

Our data showed a significant increase in the TLR4/MyD88/NF-κB pathway, accompanied by elevated levels of the proinflammatory cytokines TNF-α, IL-1β, and IL-6 compared to the control group, supporting previous studies [61,62,63,65]. Conversely, intraperitoneal injection of NAC with 5-FU significantly reduced the immunoexpression and fold changes of the TLR4/NF-κB pathway, with a concurrent decrease in the release of proinflammatory mediators. This finding aligns with previous work by Badr et al. [66], which recognized the protective effect of NAC against cisplatin-induced renal damage through its downregulation of the TLR4/inflammasome pathway. Additionally, a study by Ryu et al. [67] demonstrated the ameliorative effect of NAC against airway inflammation in cystic fibrosis patients through its inhibition of NF-κB nuclear translocation. This suggests that NAC inhibits the TLR4/NF-κB pathway by modulating upstream regulators, such as TAK1 (TGF-beta-activated kinase 1), which is involved in TLR4 signaling and NF-κB activation. Moreover, NAC’s antioxidant properties can reduce oxidative stress, which is known to activate the TLR4/NF-κB pathway. By decreasing ROS levels, NAC may prevent the activation of redox-sensitive transcription factors like NF-κB, thereby reducing inflammation. Another potential mechanism involves the inhibition of MAPK (mitogen-activated protein kinase) signaling pathways, which are upstream regulators of NF-κB and play a role in inflammatory responses. NAC has been shown to inhibit MAPK activation, augmenting its anti-inflammatory effects [66,68].

The suppressive effect of NAC on the TLR4/NF-κB pathway is suggested as one of its cardioprotective mechanisms against 5-FU-induced cardiotoxicity.

In the present study, 100 mg/kg of 5-FU upregulated the immunoexpression of caspase-3 and the protein level of Bax, a proapoptotic marker, while decreasing the supernatant content of the antiapoptotic marker Bcl-2 compared to control animals, consistent with previous studies [69,70]. The apoptotic effect of 5-FU can be attributed to its activation of ROS production and the overproduction of inflammatory cytokines. Conversely, NAC coadministration downregulated Bax and caspase-3 and upregulated Bcl-2, in line with findings by Wu et al. [71]. NAC’s antiapoptotic properties offer an additional protective mechanism against 5-FU-induced cardiotoxicity, likely due to its mitigating effect on cardiac oxidative stress and inflammation. One of the primary limitations of our study is that the dosages used were optimized for rats and may not directly translate to human dosages. Another limitation is that human physiological and pathological conditions are more complex due to comorbidities, lifestyle, and genetic variability.

## 5. Conclusions

In summary, the present study demonstrated the cardioprotective effect of NAC against 5-FU-induced cardiotoxicity, as evidenced by improvements in cardiac enzymes and the histological appearance of cardiac tissues. The cardioprotective effect of NAC is attributed to its upregulation of antioxidant enzymes via activation of the Nrf2/HO-1 pathway. Additionally, NAC inhibited the cardiac inflammatory response by downregulating the TLR4/NF-κB pathway. Moreover, it reduced cardiomyocyte apoptosis by inhibiting caspase-3 and Bax while stimulating Bcl-2. These findings suggest the potential for clinical application of NAC against 5-FU-induced cardiac impairment.

## Data Availability

The data that support this research will be shared upon reasonable request to the corresponding authors.
